# Colocalization by cross-correlation, a new method of colocalization suited for super-resolution microscopy

**DOI:** 10.1186/s12859-024-05675-z

**Published:** 2024-02-02

**Authors:** Andrew D. McCall

**Affiliations:** Optical Imaging and Analysis Facility, School of Dental Medicine, University at Buffalo, Buffalo, NY USA

**Keywords:** Colocalization, Image analysis, Image cross-correlation spectroscopy, Cross-correlation, Super-resolution

## Abstract

**Background:**

A common goal of scientific microscopic imaging is to determine if a spatial correlation exists between two imaged structures. This is generally accomplished by imaging fluorescently labeled structures and measuring their spatial correlation with a class of image analysis algorithms known as colocalization. However, the most commonly used methods of colocalization have strict limitations, such as requiring overlap in the fluorescent markers and reporting requirements for accurate interpretation of the data, that are often not met. Due to the development of novel super-resolution techniques, which reduce the overlap of the fluorescent signals, a new colocalization method is needed that does not have such strict requirements.

**Results:**

In order to overcome the limitations of other colocalization algorithms, I developed a new ImageJ/Fiji plugin, Colocalization by cross-correlation (CCC). This method uses cross-correlation over space to identify spatial correlations as a function of distance, removing the overlap requirement and providing more comprehensive results. CCC is compatible with 3D and time-lapse images, and was designed to be easy to use. CCC also generates new images that only show the correlating labeled structures from the input images, a novel feature among the cross-correlating algorithms.

**Conclusions:**

CCC is a versatile, powerful, and easy to use colocalization and spatial correlation tool that is available through the Fiji update sites. Full and up to date documentation can be found at https://imagej.net/plugins/colocalization-by-cross-correlation. CCC source code is available at https://github.com/andmccall/Colocalization_by_Cross_Correlation.

**Supplementary Information:**

The online version contains supplementary material available at 10.1186/s12859-024-05675-z.

## Background

Many methods of colocalization, particularly the most commonly used ones, rely on direct one-to-one pixel-wise (a term analogous to pointwise in mathematics) comparisons of corresponding pixels between two fluorescent images [1, 2]. These fluorescent images can be acquired using numerous different types of microscopes, different methods, and different parameters of image acquisition. Importantly, everything about the imaging conditions, from the choice of microscope to the objective, camera, and even the mounting media used during sample preparation, influence the resulting fluorescence image, particularly the resolution.

These pixel-wise colocalization techniques effectively quantify the degree of overlap of two fluorescent images. When the corresponding pixel intensities strongly correlate across the two images, typically measured using the Pearson Correlation Coefficient (PCC) or a similar correlation metric [1–4], the signals are said to be colocalized. However, since the images being quantified are dependent upon the resolution of the imaging system, the result of pixel-wise colocalization quantification is as well [1]. If image resolution is improved with the use of a better imaging system, the overlap between the two images will become smaller, as no two proteins can occupy the same physical space, resulting in lower correlation coefficients. Put simply, as better microscopes and better practices, such as using appropriate mounting media or applying deconvolution, are used, these colocalization results generally get worse, not better. For example, when the resolution becomes narrower than the distance between the two fluorophores, many colocalization algorithms will show no correlation. As new super-resolution microscopes and imaging techniques continue to push the boundaries of obtainable resolutions, these traditional pixel-wise colocalization methods will become increasingly obsolete.

Furthermore, the methods in most manuscripts only provide enough details to potentially determine the theoretical resolution limit, which can be substantially different than the obtained resolution [5, 6]. This means that the interpretation and evaluation of pixel-wise colocalization analyses can be unreliable. Colocalization analyses also suffer from many other possible hazards, such as high autofluorescence or failing to use a mask to exclude background pixels, that can result in false positives or false negatives [5].

As an alternative, the cross-correlation function (CCF) can be used to evaluate spatial correlation between two fluorescent images, a process commonly referred to as image cross-correlation spectroscopy (ICCS) [7–15]. ICCS has the distinct advantage of providing results that improve with increasing resolution. In this methodology, rather than analyzing only the direct overlap of fluorescent images, the images are shifted relative to one another and their correlation is re-evaluated across a range.

ICCS methods offer several advantages over the direct overlap methods. Most importantly, there is no requirement for ICCS methods to have any overlap, allowing for correlation at a distance greater than the resolution. This makes ICCS methods significantly better for super-resolution microscopy techniques, where it is more likely that the resolution will be great enough that traditional pixel-wise colocalization techniques will fail. Additionally, changes to the resolution of the analyzed images are reflected in the resulting correlation curve when using ICCS methods. This means that efforts to improve the resolution of images will lead to better, more precise results.

The ICCS plugin presented here, Colocalization by Cross-Correlation (CCC), is conceptually similar to van Steensel’s CCF and other ICCS algorithms, but with several improvements to increase its utility and versatility. First, instead of being limited to a short range or two-dimensional images, cross-correlation is determined across the entire image in all three spatial dimensions. Second, this plugin uses pixel randomization to remove the contribution of low spatial frequency repeating elements (e.g., the repetitive pattern of nuclei grown in monoculture) and to provide an estimate of statistical confidence. Third, after the removal of the low spatial frequency repeating elements, a Gaussian curve is fitted to the data, providing accurate mean (µ) and standard deviation (σ) distance values for the spatial correlation. This Gaussian curve data is then used to construct two signal contribution images, which highlight the specific fluorescent signal that contributed the most to the Gaussian curve result from both input images, while suppressing data that did not contribute.

CCC generates two statistical parameters that can be used to evaluate the results. Confidence, as mentioned previously, is generated via a pixel randomization method; it serves a similar purpose to the Costes significance test of traditional pixel-wise colocalization methods [3, 16], which is to determine if the signal is so homogenous in either input image that a positive colocalization result is inevitable. In CCC, confidence values range from 0 to 1. A zero value represents low confidence, a result that likely arises from either a lack of a true spatial correlation between the images or high autocorrelation (measurable using a normalized autocorrelation function) in at least one of the images. High autocorrelation is the result of the molecular density being too high or the resolution being too low (see Additional file 1: Figure S1). A confidence value of one suggests the result is very likely indicative of a true spatial correlation. CCC also reports an R^2^ value calculated from the Gaussian curve fit.

Here, to establish the validity of the plugin and the confidence metric, the effects of numerous parameters of spatial correlation (i.e., correlation distance, molecular density and non-correlating signal) and image quality (i.e., resolution, background, and noise) are tested with the CCC plugin. Artificial 3D test data was used so that the parameters could be precisely adjusted. CCC proved highly robust, able to identify the correct spatial correlation in many challenging cases, including over large distances, in the presence of excessive noise, and with eightfold additional non-correlated points in each image. Additionally, improvements to image resolution improved the accuracy and precision of the CCC results. Overall, the data demonstrates that CCC is a useful plugin for colocalization and spatial correlation analyses.

## Implementation

### Image pre-processing

Prior to analysis a few image pre-processing steps are required for CCC to function as intended. As illustrated in Table 6, the presence of even low mean values of background, measured as the pixel intensity in regions devoid of signal, results in a very low confidence value. As most image formats cannot support negative values, simply subtracting the mean background value will still result in a positive average mean background and low confidence (see Table 9). Thus, the image format must first be changed to one that supports negative values (e.g. 32-bit command in ImageJ) prior to subtracting the mean background value. When done in this manner, the confidence value from CCC properly represents the likelihood that a result is from a true spatial correlation.

Additionally, a segmented image mask must be prepared that divides the image into foreground and background for the algorithm to work properly. Generally, the foreground of this mask should define all possible localizations for one of the labeled structures to be analyzed. For example, if one is determining possible spatial correlation between two nuclear proteins, then the foreground would be the nuclei. The mask is important for the appropriate function of the plugin, as it has several functions, detailed below, that can heavily influence the results. Using an inappropriate mask often leads to very large σ values with a µ of zero, or very low confidence.

Lastly, to generate accurate distance measurements, the image scaling metadata must be present and accurate for all dimensions.

### Determining spatial correlation

A graphical abstract of all CCC processing steps described below can be found in Additional file 1: Figure S2.

**Fig. 1.**
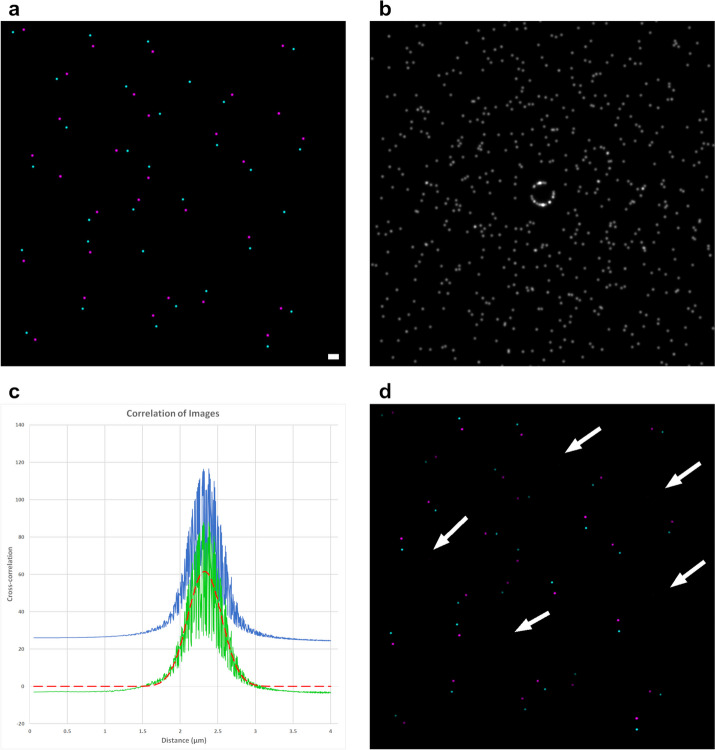
2D example data of CCC plugin results. **a** Color composite of the two input images, with most of the magenta points being paired to a corresponding cyan point. Scale bar indicates 2.32 µm for all images, the returned value of µ after analysis. **b** Image generated from the cross-correlation of the input images. In this cross-correlated image, the center pixel value represents the correlation between the two colors with no translation, and all other pixels represent the correlation when one image has been translated by a vector from the center to that pixel. The ring near the center of the image matches the paired points, with gaps indicating a lack of pairs in that orientation. **c** The blue line is the radial profile of **b**, the green line is the radial profile after the pixel randomization procedure (see Implementation for details), and the red dashed line is the fit Gaussian curve. **d** Composite image of the data that contributed to the Gaussian result. Arrows indicate location of signal from the original images that did not contribute

Before any cross-correlation, all pixel intensities outside of the masked regions are set to zero for both images so that these pixels will not contribute to the result. Then, the cross-correlation between the two mask-modified input images is determined using the ImgLib2 convolution algorithm [17] (for more details on this algorithm see Additional file 1), producing a new image of the cross-correlation result (CCR) where a high pixel intensity indicates a point of correlation (Fig. 1b). The distance from the center of the CCR to the point of correlation indicates the distance of the spatial correlation. At this point, the CCR shows all correlations that exist between the two images, including those from low spatial frequency repeating elements (e.g., nuclei that are spaced roughly evenly apart in a cell culture monolayer) that we may not wish to have as our result.

Thus, the next step is to subtract out from the CCR image the contribution of low spatial frequency repeating elements. To start this, the first input image is subjected to a pixel randomization, where pixels within the mask region are randomly re-assigned to another location within the mask region. The only way this process differs from Costes randomization is that individual pixels are randomized as opposed to point spread function (PSF; the 3D diffraction pattern from a point source [1])-sized blocks [3]. Pixel randomization was used as the implementation was much simpler, less error-prone and allowed for more precision in relation to the mask provided. Extensive testing showed that pixel randomization versus Costes randomization made no difference in the results of CCC (data not shown). The randomized image is then cross-correlated with the unmodified second input image to produce a non-specific cross-correlation image. The process of randomization and cross-correlation is repeated a number of times defined by the user, and the average of all the non-specific cross-correlation images is taken. This repeated randomization and averaging process is the reason that pixel randomization and Costes randomization produce nearly identical results, as they both trend towards the same averaged non-specific correlation image over repeated randomizations. This averaged non-specific correlation image is then subtracted from the CCR to produce a subtracted cross-correlation result (SCCR). In the SCCR, contributions from any low spatial frequency repeating elements should be almost entirely removed, assuming an adequate mask is used. Additionally, as the total intensity in the randomized image equals the total intensity of the original image, the average correlation across the SCCR image will be near zero, with only specific spatial correlations in the original CCR image remaining as strong correlations. To determine the spatial correlation’s mean distance and standard deviation, a Gaussian curve is then fitted to the radial profile of the SCCR image (Fig. 1c). During the generation of the radial profile, the image scaling metadata for all dimensions is used to ensure accurately reported distances. A Gaussian curve is used because they approximate the shape of a PSF. If a spatial correlation does not exist between the input images, the curve fitting process may fail. Curve fitting failures are rare since CCC makes every attempt to fit a gaussian curve somewhere within the data, but they can occur when the input images are abnormally smooth (e.g. an unstained negative control) or when bad, overly restrictive masks are used. When curve fitting does fail error results are given for CCC’s output, with confidence and R^2^ set to − 1.

We then need to establish our statistical measures. First, the coefficient of determination (R^2^) within three standard deviations of the mean spatial correlation is calculated for our Gaussian fit model. This value will change largely based on the noise within the image, as well as the degree of anisotropy of the PSF. Next, a confidence value is determined by calculating the fraction between the integral of the SCCR radial profile (SCCR_RP_) and the CCR radial profile (CCR_RP_) within three standard deviations of the mean spatial correlation, as such:

$$C\left( d \right) = \frac{{\mathop \smallint \nolimits_{ - 3\sigma + \mu }^{3\sigma + \mu } SCCR_{RP} \left( d \right)}}{{\mathop \smallint \nolimits_{ - 3\sigma + \mu }^{3\sigma + \mu } CCR_{RP} \left( d \right)}}$$

where $d$ denotes the distance of cross-correlation, and µ and σ represent the mean and standard deviation of the Gaussian fit. The confidence will decrease when there is high background relative to the signal, when the autocorrelation is high in either input image, when there is significant real signal present in either image that is not within 3 standard deviations of µ, or if a true spatial correlation does not exist within the image and a Gaussian curve was fit to a random correlation within the data. For this purpose, autocorrelation can be measured as the full area under the curve of the autocorrelation radial profile.

### Constructing the contribution images

One strength of the pixel-wise methods over cross-correlation methods has been the ability to easily visualize the fluorescent signal that contributed to the result. Accomplishing this with the direct overlap methods is fairly straightforward because each data point in the scatterplot used to evaluate the PCC directly corresponds to a specific pixel location in the fluorescent images. With ICCS methods, determining which part of the fluorescent signal from each input image contributed to the result is more challenging. This is because every pixel in both input images contributes to all pixels in the CCR image (i.e., not a one-to-one relation). To determine the signal contribution images, we have to effectively reverse the operations, using the cross-correlation result and the original images to mathematically calculate the contribution images.

To begin this process, the Gaussian fit curve is used to modify the SCCR image. First, every pixel in the SCCR image has its distance to the center of the image determined, and then the pixel value is multiplied by the value of the Gaussian curve at that distance, creating a new Gaussian-modified CCR (GCCR) image. In the GCCR image, all the pixel values are suppressed except those at the distance of the spatial correlation, which is why this image is used to mathematically work backwards to create the contribution images. The exact functions used to construct the two contribution images are slightly different from one another, and are dependent on the order used in the original cross-correlation. If we take the function:

$$CCR = img1 \star {\text{img2}}$$

where ⋆ is the correlation operator, then we can evaluate the contribution of img1 to the Gaussian fit result (Cont_img1_) using the equation:

$$Cont_{img1} = \left( {img2*GCCR} \right) \cdot {\text{img}}1$$

where ∗ is the convolution operator, and · is the pixel-wise multiplication operator. Conceptually, this function first creates a new image (img2 * GCCR) that effectively highlights all possible points that are within the spatial correlation distance of any fluorescent signal in img2. This means that any spatially correlated signal in img1 should be found within the signal of the (img2 * GCCR) image. Thus, to isolate the contribution of img1, we only need to multiply each pixel of img1 to the corresponding pixel in the (img2 * GCCR) image. Once complete, this produces a new image of img1 whose brightest components are those within the µ ± σ range of signal in img2 (Fig. 1d). Importantly, the relative intensities in the contribution image depends not only on the distance of the spatial correlation, but also its orientation, which is exactly why some of the contributing pairs in Fig. 1d are brighter than others, as they had matching orientations with other pairs within the images.

Obtaining the contribution of img2 is very similar, and is determined using the equation:

$$Cont_{img2} = \left( {img1 \star GCCR} \right) \cdot {\text{img2}}$$

The reason a correlation operation is performed in place of the convolution operation is because the orientation of the spatial correlation is mirrored. Thus, the GCCR needs to be mirrored during the calculations, and this is exactly what the correlation operation does.

## Evaluation

### PSF image acquisition

PSF images for widefield (WF), spinning-disk 40 µm pinhole (SD-40), and spinning-disk 25 µm pinhole (SD-25) were acquired via imaging 0.2 µm tetraspeck beads (ThermoFisher, T7280) mounted in optical cement using an Andor Dragonfly Spinning Disc confocal microscope with a 40x/1.3 oil objective at 520/40 nm and 592/25 nm emissions and imaged on a Zyla 4.2 sCMOS camera with an additional 2 × camera magnification, unless otherwise specified.

WF and SD Deconvolution was completed by 10 iterations of the Richardson-Lucy algorithm in Deconvolution Lab 2 version 2.1.2 using theoretical PSFs created with the PSF Generator plugin [18, 19]. PSFs were generated using the Born & Wolf model, with parameters matching the acquisition parameters: best accuracy setting, 1.5 immersion refractive index, 1.3 NA, 75.39 nm pixel size and z-step, 128 × 128 × ﻿65 output diameters, and both 520 nm and 592 nm wavelengths. Though not usually recommended, theoretical PSFs were chosen instead of acquired PSFs as using the same, or nearly identical, image for deconvolution that was used to convolve the test images in the first place would be an unrealistic standard when compared to traditional biological imaging.

iSIM PSFs were acquired via imaging 0.1 µm tetraspeck beads (ThermoFisher, T7279) on a VT-iSIM (VisiTech International) modeule with Ingwaz scanning architecture attached to a Leica DMi8 inverted microscope stand with a HCPLAPO 100x/1.4 oil objective. The beads were excited at 488 and 561 nm, and emission was captured using two Hamamatsu Orca fusion sCMOS cameras at 525 nm and 595 nm emission wavelengths. The iSIM pinhole array was set to 50 µm during imaging. Raw iSIM images were deconvolved using the VisiView (VisiTech International) embedded Microvolution v2022.10.0.0 software through 10 iterations of blind deconvolution using default settings and an auto generated PSF from the image metadata. Lastly, a small chromatic shift between channels was corrected for using Fijiyama version 4.2.2 with block-matching rigid registration and default parameters [20].

3D Stimulated emission depletion microscopy (STED) PSFs were acquired via imaging 0.12 µm abberior nanoparticles (NP-3011) using an abberior Facility Line STED module attached to an Olympus IX83 microscope stand with a UPLXAPO 100x/1.45 oil objective. Pulsed excitation laser lines of 561 nm and 640 nm were used with a 775 nm pulsed depletion laser at 30% and 25% depletion power for each excitation laser, respectively. Emission was captured at 600/60 nm and 702/105 nm on Avalanche photodiode detectors, and an Adaptive Optics deformable mirror was used to correct for spherical aberrations. Images were acquired with the 3D STED depletion pattern at 100% to generate near isotropic PSFs. The 0.12 µm beads used for STED imaging were slightly too large for ideal PSF generation, but still represent a substantial resolution improvement over WF and SD images.

All PSFs were imaged to meet the Nyquist sampling criterion (minimum 2.5 pixels per PSF) in both the lateral and axial directions. Following acquisition, all PSFs were digitally resampled to be isometric using the ImageJ reslice command, which was necessary as all paired points in the test images were generated assuming isometric scaling.

### Test data

Artificial 3D test images composed only of single foreground pixels were created where points were paired across two images with a set distance between pairs and a random orientation. The location and orientation of the paired points was randomized in every generated test image. The base artificial test images, for the 40x/1.3NA objective SD PSF data at 1.13 µm spatial correlation distance (SCD), were generated with image dimensions of 512 × 512 × 128 pixels and a spacing between paired foreground pixels of 15 pixels. For all other PSF data the image dimensions and pixel spacing were adjusted to compensate for the difference in voxel dimensions of the PSF to generate the same final scaled image volume and scaled spacing between particles. For Table 5, additional uncorrelated randomly localized points were added to each image. These base images were then convolved with isolated PSFs from the PSF images described above to produce scaled images of correlated PSFs. Where indicated, additional background or Gaussian distributed noise of the indicated standard deviation was added across the entire image. The noise was Gaussian distributed to simulate dark noise and readout noise. Unless otherwise indicated, test images were generated at a molecular density of 3.48 × 10^–3^ particles/µm^3^ within a total volume of 14,345 µm^3^ using deconvolved SD-40 PSFs.

Test images were then analyzed for spatial correlation using the CCC plugin with 10 cycles of pixel randomization. The process of test image generation and analysis was repeated 10 times for each condition tested, and all results are reported as the mean and standard deviation (SD) of the obtained plugin results. The percent accuracy of the average µ value was then evaluated using the formula:

$$Accuracy = 100 - \frac{{\left| {\overline{\mu } - SCD} \right|}}{SCD} \times 100$$

For PCC analysis, the test images were analyzed using the Fiji plugin Coloc 2, and the Pearson’s R value with no threshold was reported. No thresholding was used as the Costes threshold results were inconsistent across repeats.

### Fluorescent staining

MLE-12 cells [21] (American Type Culture Collection CRL-2110) were seeded on coverslips (Fisherbrand 12-545-82 12CIR-1D) at a density of 50,000 cells/well. Twenty-four hours after seeding, cells were incubated with MitoTracker™ Red CMXRos (ThermoFisher M7512), 150 nM for 15 min, fixed with ice-cold 100% methanol for 15 min, and ER was visualized using anti-protein disulfide isomerase (PDI) antibodies. Briefly, cells were permeabilized with 0.05% saponin-PBS, non-specific staining was blocked by incubation with 5% goat serum in 0.05% saponin-PBS for 30 min at room temperature, mouse anti-PDI antibodies (Enzo Life Sciences ADI-SPA-891-D, 1:1000) were added for 2 h at room temperature, washed three times, incubated with secondary antibodies conjugated to Alexa Fluor 488 (Invitrogen A-11029) for 1 h at room temperature, and washed twice with 0.05% saponin-PBS. Nuclei were visualized by Hoechst 33,258, (Sigma-Aldrich 14,530), cells were washed once with 0.05% saponin-PBS, and once with PBS, and mounted using Prolong Gold Antifade mounting medium (ThermoFisher P36930).

Cells were imaged using a Leica SP8 confocal microscope with a 63x/1.4 oil objective, 48.3 nm pixel size, 299 nm z-steps, and a 1 Airy unit pinhole. Cells were excited sequentially at 405, 488 and 572 nm and emission was captured at 460/90, 536/25, and 620/72, respectively. The 3D images were then deconvolved through 25 iterations of the Richardson-Lucy algorithm in Deconvolution Lab 2 using PSFs acquired from 0.2 µm tetraspeck beads under the same imaging conditions [18]. Deconvolved images were then then analyzed for spatial correlation using the CCC plugin with 10 cycles of pixel randomization and an analysis mask. To generate the mask, a copy of the ER image was blurred using the ImageJ Gaussian blur 3D command with 2 pixel radius for all axes, and thresholding was then applied using the Otsu method to create a mask of the cytoplasm [22]. A mask of the nucleus, generated the same way, was then subtracted from the cytoplasm mask to ensure no region within the nucleus was included in the analysis.

## Results

### Example of CCC results

Figure 1a shows a simple demonstrative 2D example input image for the CCC plugin. This type of non-overlapping relation shows no correlation when using any pixel-wise colocalization methods. However, by analyzing the cross-correlation of the input images (Fig. 1b) CCC correctly identifies a spatial correlation with a µ of 2.32 µm and a σ of 0.23 µm (Fig. 1c). It also calculates which points in the original images contributed to this result (Fig. 1d).

### Effect of imaging modalities

The choice of imaging modality greatly influences the results of nearly any microscopy image analysis tool. Thus, the effects of imaging modality choice were tested. Widefield (WF), spinning disc confocal (SD-40 and SD-25 for 40 µm and 25 µm pinholes, respectively), iSIM, and STED imaging modalities were tested, as well as the effects of subsequent deconvolution. The 3D input images were artificially generated from real PSF images, and all had a true SCD of 1.13 µm (see Additional file 2 for example). Exposure times were adjusted as needed, but all other imaging parameters were kept the same where possible.

Unsurprisingly, it was found that WF imaging produced the least accurate results (Table 1), with one repeat producing a µ value of 0 µm and most of the remaining producing µ values in the range of 0.83–0.93 µm. WF imaging has the greatest difference between its lateral and axial resolutions, making it more sensitive to the orientation of the paired PSFs. The results for confocal imaging were slightly improved, but more consistent, with no repeats producing µ values of zero. It was expected to see an improvement of SD-25 results over SD-40; however, SD-25 PSF images had more noise, with only a slight improvement in z-axis resolution. Thus, the difference in results between the two was negligible.

**Table 1:** Effect of imaging modality on CCC results

Imaging modality	µ (µm)	σ (µm)	Confidence	R^2^
WF	0.83 (0.29)	1.27 (0.40)	0.42 (0.07)	0.70 (0.11)
SD-40	0.89 (0.02)	0.53 (0.04)	0.73 (0.01)	0.74 (0.08)
SD-25	0.89 (0.02)	0.58 (0.07)	0.63 (0.03)	0.68 (0.07)
Decon WF	1.01 (0.03)	0.32 (0.02)	0.79 (0.01)	0.65 (0.09)
Decon SD-40	1.05 (0.02)	0.26 (0.02)	0.87 (0.01)	0.47 (0.12)
Decon SD-25	1.07 (0.01)	0.24 (0.01)	0.81 (< 0.01)	0.57 (0.15)
iSIM	1.04 (0.02)	0.28 (0.02)	0.92 (< 0.01)	0.57 (0.13)
3D-STED	1.09 (0.01)	0.19 (0.01)	0.94 (< 0.01)	0.41 (0.15)
True value	1.13			

PSFs collected from various microscope imaging modalities were used to generate input images for CCC with 50 spatially correlated pairings at 1.13 µm correlation distance. Results from the CCC plugin for each imaging modality are shown. All values are shown as Mean (SD) for n = 10 repeats

Deconvolution of the input images improved the results substantially, with the deconvolved WF PSF producing more accurate and precise results than the non-deconvolved confocal PSFs. Deconvolving the SD-40 and SD-25 PSFs improved the results even further, to an accuracy of 94.7% for the deconvolved SD-25 images. Deconvolution also caused a decrease in the R^2^ value, this appears to be the result of an increase in the relative brightness of camera noise around the PSF compared to the peak brightness of the PSF. However, this decrease was relatively minor, and likely enhanced by how the test images were generated.

Deconvolved iSIM images produced µ and σ values similar to deconvolved SD-40 images, though with improved confidence and R^2^ values. While improved µ and σ results with iSIM images were initially expected, the resolution increase in the iSIM images compared to the SD-40 images was only in the lateral axis, with no improvement in the optical axis. Additionally, the lateral resolution increase for the red channel was relatively small, and this channel had some mild aberrations. The combination of these factors seems to be responsible for the similar results observed.

Super-resolution 3D-STED imaging improved nearly all the results compared to SD and iSIM, producing the highest accuracy for the µ value (96.5%) and reducing the σ values to below 200 nm. Additionally, due to the low autocorrelation of the high resolution images, the confidence values were very close to 1. These STED results could have been even better with a more optimal sample for imaging, but they still clearly demonstrate that super-resolution imaging with STED generates improved results with CCC. Deconvolution was not performed on the STED images as an appropriate theoretical PSF could not be generated using the available tools. This data clearly demonstrates that CCC results improve when higher resolution imaging modalities are used.

### Effect of numerical aperture and magnification

The objective lens is largely what defines the resolution limit of a microscope; thus, picking the best objective for your experiment is critical. Given the importance of resolution to colocalization analysis, multiple objective PSFs were tested on CCC results. Due to the lower numerical aperture (NA) of many of the objectives tested, the non-deconvolved SD-40 microscope configuration was used for collection of the PSFs in Table 2, as SD-25 resulted in too much signal loss. The NA is the primary factor that determines the resolution limit of an objective. All test images were generated to be the same total volume and molecular density.

**Table 2:** Effect of objective lens selection on CCC results

Magnification/NA	µ (µm)	σ (µm)	Confidence	R^2^
20x/0.75	0.03 (0.08)	4.48 (0.60)	0.14 (0.02)	0.57 (0.05)
25x/0.95	0.04 (0.08)	2.30 (1.07)	0.34 (0.09)	0.71 (0.10)
40x/1.1	0.90 (0.05)	0.74 (0.17)	0.65 (0.04)	0.72 (0.04)
40x/1.3	0.88 (0.02)	0.53 (0.05)	0.73 (0.02)	0.77 (0.09)
63x/1.3	0.90 (0.02)	0.50 (0.02)	0.82 (0.01)	0.80 (0.07)
True value	1.13			

Non-deconvolved PSFs collected using various objectives were used to generate input images for CCC with 50 spatially correlated pairings at 1.13 µm correlation distance. Results from the CCC plugin for each objective used are shown. All values are shown as Mean (SD) for n = 10 repeats

As shown in Table 2, both the 20x/0.75 NA and 25x/0.95 NA objectives resulted in µ values at or near zero (< 3% accuracy), and large σ values. Additionally, due to the high autocorrelation these objectives produced results with low confidence. Under ideal circumstances, these objectives should have been sufficient to return non-zero µ values, however both of these objectives showed aberrations in their PSFs that decreased their resolution. Once a sufficiently high NA objective that didn’t produce aberrations was used, the µ value settled around 0.9 µm, with increasing resolution and magnification resulting in improved σ, confidence, and R^2^ values. This further demonstrates that CCC results improve as image resolution improves, and that a critical resolution must be met to determine non-zero spatial correlations.

### Effect of correlation distance

A strength of the CCC plugin is its ability to analyze spatial correlations at distances greater than the resolution limit of the imaging system. However, differences in the true SCDs will impact more than just the µ value, even with all other things being equal. The effects of various true SCDs on the output of the CCC plugin compared to PCC results are shown in Table 3. It was found that true SCDs near the resolution limit of the imaging system, where PCC results indicate colocalization, would nearly always result in µ values of zero, with σ values steadily increasing instead as the true SCD increased. Beyond a SCD threshold (around 0.35 µm for the deconvolved 40x/1.3 SD-40 PSF used here), non-zero µ results became consistent, while the PCC values decreased substantially, indicating no colocalization. As the true SCD increased substantially more, the CCC results actually became more accurate (99% accuracy at 2.26 µm SCD), though with a slight increase in σ when compared to shorter distances. The most notable difference with increasing SCD was a drop in the statistical measures, both confidence and R^2^, though this remained reasonably high for all distances tested and well within the range for positive results. These data suggest that a critical resolution exists for any given spatial correlation distance that if not met will yield zero-valued µ results. This resolution cut-off is approximately the average of the axial and lateral full-width half-maximums of both PSFs (0.34 µm) for the randomly oriented data shown here.

**Table 3:** Effect of particle distance on CCC results

True distance (µm)	µ (µm)	σ (µm)	Confidence	R^2^	PCC
0	0 (0)	0.20 (< 0.01)	0.99 (< 0.01)	0.84 (0.01)	0.79 (< 0.01)
0.3	0.02 (0.05)	0.27 (0.02)	0.98 (< 0.01)	0.85 (0.04)	0.22 (0.03)
0.45	0.34 (0.03)	0.21 (0.02)	0.96 (< 0.01)	0.81 (0.06)	0.06 (0.01)
0.6	0.49 (0.01)	0.23 (0.01)	0.94 (< 0.01)	0.72 (0.13)	0.02 (< 0.01)
1.13	1.06 (0.02)	0.24 (0.02)	0.81 (0.01)	0.54 (0.11)	0 (< 0.01)
2.26	2.24 (0.02)	0.32 (0.07)	0.55 (0.01)	0.33 (0.08)	0 (< 0.01)

Deconvolved SD-40 PSFs collected with a 40x/1.3 objective were used to generate input images for CCC with 50 spatially correlated pairings at various correlation distances. Results from the CCC plugin and Persons R value (PCC) for each distance are shown. All values are shown as Mean (SD) for n = 10 repeats

The observed slight increase in the σ value with increasing true SCD appears to be a result of an increase in contribution from non-paired particles at greater distances. This can be explained because the volume of the spherical shell defined by µ ± σ increases as µ increases, even with a fixed σ value. Thus, the probability that a non-paired particle would be within or near enough to this spherical shell during the cross-correlation increases as well, causing a slight increase in σ as these non-paired particles influence the results. These non-paired particles also cause deviations in the cross-correlation profile away from the fit Gaussian curve, resulting in a drop in R^2^.

### Effect of molecular density

Generally, the molecular density of any given target molecule within a biological sample will vary significantly compared to any other target. Therefore, the effects of various numbers of correlated PSF pairs within the same volume was tested with CCC (see Additional File 1: Figure S3 for an example image). As shown in Table 4, while increasing molecular density of correlated pairs causes a corresponding drop in confidence, it did not have a significant effect on other parameters until a high critical density, at which point the σ value increased significantly, likely due to the high autocorrelation. Since proteins tend to have more order than the randomly positioned molecules in our test images, it is likely that biological data will have a higher critical density cutoff.

**Table 4:** Effect of molecular density on CCC results

Particles/µm^3^	µ (µm)	σ (µm)	Confidence	R^2^
3.48 × 10^–4^	1.07 (0.01)	0.25 (0.02)	0.81 (0.01)	0.52 (0.15)
1.39 × 10^–2^	1.07 (0.01)	0.25 (0.01)	0.52 (0.01)	0.54 (0.08)
5.58 × 10^–2^	1.07 (0.01)	0.26 (0.01)	0.21 (< 0.01)	0.55 (0.04)
0.22	1.07 (0.01)	0.26 (0.02)	0.06 (< 0.01)	0.56 (0.02)
0.87	1.07 (0.01)	0.28 (0.03)	0.02 (< 0.01)	0.58 (0.04)
1.74	1.08 (0.02)	5.71 (6.81)	0.01 (< 0.01)	0.45 (0.18)
True value	1.13			

Deconvolved SD-40 PSFs collected with a 40x/1.3 objective were used to generate input images for CCC with various numbers of spatially correlated pairings, reported as molecular density per image, at 1.13 µm correlation distance. Results from the CCC plugin for each molecular density are shown. All values are shown as Mean (SD) for n = 10 repeats

### Effect of additional non-correlated particles

It would be exceedingly rare in biology to have any two fluorescent probes exhibit perfect 1:1 pairing or perfect compartmentalization. Proteins are regularly trafficked to different organelles, and protein binding is often dependent on cell signaling or environmental conditions. Thus, it is important that any colocalization assay can positively identify spatial correlation even in the presence of non-correlating signal. This was tested with the CCC plugin by adding additional randomly positioned unpaired points to both input images (see Additional file 1: Figure S3 for an example image). The base image had a paired particle density of 3.48 × 10^–4^/µm^3^.

CCC proved capable in this regard, being able to positively identify true spatial correlation even when each input image had an additional 2.79 × 10^–2^/µm^3^ randomly placed particles, equaling eight times as many uncorrelated points as correlated points (Table 5). Due to the high autocorrelation, the confidence in such a result was quite low, only 0.04 on average, and the µ and σ values were less consistent across repeated tests compared to results with fewer uncorrelated points. The R^2^ value also decreased with increasing non-correlated signal, though not as drastically as the confidence. However, despite these low values for statistical measures, CCC still obtained µ values that were within one σ of the true SCD for every repeated test, and was 84% accurate on average. Additionally, all tests with fewer than 2.79 × 10^–2^/µm^3^ additional non-correlated particles had results nearly as accurate as the images with no additional points (> 93% accuracy), though with expected decreases in the statistical measures, confidence and R^2^.

**Table 5:** Effect of additional non-correlated particles on CCC results

Extra particles/µm^3^	µ (µm)	σ (µm)	Confidence	R^2^
0	1.07 (0.02)	0.25 (0.01)	0.81 (0.01)	0.57 (0.10)
6.97 × 10^–4^	1.07 (0.02)	0.25 (0.03)	0.75 (0.01)	0.52 (0.12)
1.74 × 10^–3^	1.06 (0.01)	0.24 (0.02)	0.65 (0.01)	0.54 (0.15)
3.49 × 10^–3^	1.07 (0.01)	0.25 (0.03)	0.51 (0.01)	0.47 (0.16)
6.97 × 10^–3^	1.07 (0.03)	0.24 (0.04)	0.30 (0.02)	0.43 (0.09)
1.39 × 10^–2^	1.08 (0.05)	0.27 (0.07)	0.13 (0.01)	0.34 (0.12)
2.79 × 10^–2^	0.95 (0.33)	1.85 (3.63)	0.04 (0.02)	0.28 (0.18)
True value	1.13			

Deconvolved SD-40 PSFs collected with a 40x/1.3 objective were used to generate input images for CCC with 50 spatially correlated pairings at 1.13 µm correlation distance. Extra particles indicate the density of additional uncorrelated PSFs that were added to each input image prior to analysis. Results from the CCC plugin for each extra particle quantity are shown. All values are shown as Mean (SD) for n = 10 repeats

### Effect of high background

The effects of camera offset, and other sources of background signal, were tested by uniformly adding value to all pixels of the test images. As seen in Table 6, even small decreases to the signal to background ratio result in a significant drop in the result confidence. This is an expected result based on how confidence is calculated (see Implementation); however, it illustrates the importance of performing a pre-processing background subtraction before using CCC.

**Table 6:** Effect of additional image background on CCC results

Signal to background	µ (µm)	σ (µm)	Confidence	R^2^
No background	1.07 (0.02)	0.25 (0.01)	0.81 (0.01)	0.47 (0.09)
1000:1	1.06 (0.02)	0.26 (0.01)	0.04 (< 0.01)	0.54 (0.12)
100:1	1.08 (0.01)	2.42 (5.41)	< 0.01 (< 0.01)	0.36 (0.14)
10:1†	8.40 (5.46)	9.03 (6.70)	< 0.01 (< 0.01)	0.33 (0.21)
True value	1.13			

Deconvolved SD-40 PSFs collected with a 40x/1.3 objective were used to generate input images for CCC with 50 spatially correlated pairings at 1.13 µm correlation distance. Additional flat background was added to each input image prior to analysis. Results from the CCC plugin for each signal to background ratio are shown. All values are shown as Mean (SD) for n = 10 repeats. † indicates that this result had two repeats fail to fit a Gaussian curve to the data and is n = 8

### Effect of noise

The noise in an image, particularly the shot noise, can never be entirely eliminated, just mitigated [23]. To see how noise affects CCC results, additional Gaussian distributed noise was added to the test images (see Additional file 1: Figure S3 for an example image). The type of noise added simulates additional dark noise and readout noise from the electronics, which affects every pixel in the image. Overall, the CCC plugin proved quite robust when faced with additional image noise (Table 7). Additional noise, up to a signal to added noise ratio (SNR) of 50:1, had seemingly no impact on the results. At a ratio of 20:1 there was a sharp drop in the R^2^ value, as would be expected, though this corresponded with only a very minor reduction in the precision of the other reported values. At a 10:1 SNR (not shown), nearly all the test images resulted in a failure to fit a Gaussian curve to the data. However, if the compounding factor of the high SCD is removed and the effect of noise at zero SCD is tested, the CCC plugin can identify correlations down to SNRs of 5:1, though at this point several repeats had µ values that deviated slightly from zero (Table 8). SNRs well above 20:1 are relatively easy to obtain in modern fluorescent microscopy techniques given reasonably bright fluorophores.

**Table 7:** Effect of additional image noise on CCC results

Signal to noise	µ (µm)	σ (µm)	Confidence	R^2^
No added noise	1.06 (0.02)	0.26 (0.01)	0.81 (0.01)	0.42 (0.11)
100:1	1.07 (0.01)	0.25 (0.02)	0.81 (0.01)	0.57 (0.14)
50:1	1.07 (0.01)	0.25 (0.01)	0.81 (0.01)	0.41 (0.14)
20:1	1.06 (0.02)	0.25 (0.02)	0.80 (0.03)	0.09 (0.02)
True value	1.13			

Deconvolved SD-40 PSFs collected with a 40x/1.3 objective were used to generate input images for CCC with 50 spatially correlated pairings at 1.13 µm correlation distance. Additional Gaussian distributed noise was added to each input image prior to analysis. Results from the CCC plugin for each signal to noise ratio are shown. All values are shown as Mean (SD) for n = 10 repeats

**Table 8:** Effect of image noise with zero spatial correlation distance on CCC results

Signal to noise	µ (µm)	σ (µm)	Confidence	R^2^
No added noise	0 (0)	0.20 (< 0.01)	0.99 (< 0.01)	0.84 (< 0.01)
20:1	0 (0)	0.20 (< 0.01)	0.99 (< 0.01)	0.84 (0.01)
10:1	0.04 (0.05)	0.18 (0.02)	0.99 (0.01)	0.78 (0.03)
5:1	0.11 (0.06)	0.13 (0.04)	1.00 (0.04)	0.43 (0.07)
True value	0			

Deconvolved SD-40 PSFs collected with a 40x/1.3 objective were used to generate input images for CCC with 50 spatially correlated pairings at 0 µm correlation distance. Additional Gaussian distributed noise was added to each input image prior to analysis. Results from the CCC plugin for each signal to noise ratio are shown. All values are shown as Mean (SD) for n = 10 repeats

It is important to note that all the noise data presented so far was based on images that allowed for negative values. If negative values are instead set to zero the results change dramatically, with confidence values rapidly decreasing with lower SNRs, similar to the effect of high background (Table 9). This occurs because zero-bounded noise will result in a non-zero mean background, thus producing similar results. This is why it is important to convert images to a signed value format prior to background subtraction during image pre-processing for CCC (see Implementation).

**Table 9:** Effect of zero-bounded image noise on CCC results

Signal to noise	µ (µm)	σ (µm)	Confidence	R^2^
No added noise	1.08 (0.02)	0.25 (0.02)	0.81 (0.01)	0.51 (0.16)
100:1	1.08 (0.02)	0.23 (0.02)	< 0.01 (< 0.01)	0.47 (0.13)
50:1	1.10 (0.05)	3.52 (5.96)	< 0.01 (< 0.01)	0.35 (0.16)
20:1	1.13 (0.09)	16.92 (32.38)	< 0.01 (< 0.01)	0.14 (0.15)
True value	1.13			

Deconvolved SD-40 PSFs collected with a 40x/1.3 objective were used to generate input images for CCC with 50 spatially correlated pairings at 1.13 µm correlation distance. Additional Gaussian distributed noise was added to each input image, then all negative values were set to zero prior to analysis. Results from the CCC plugin for each signal to noise ratio are shown. All values are shown as Mean (SD) for n = 10 repeats

### Measuring the spatial correlation between the ER and mitochondria

**Fig. 2:**
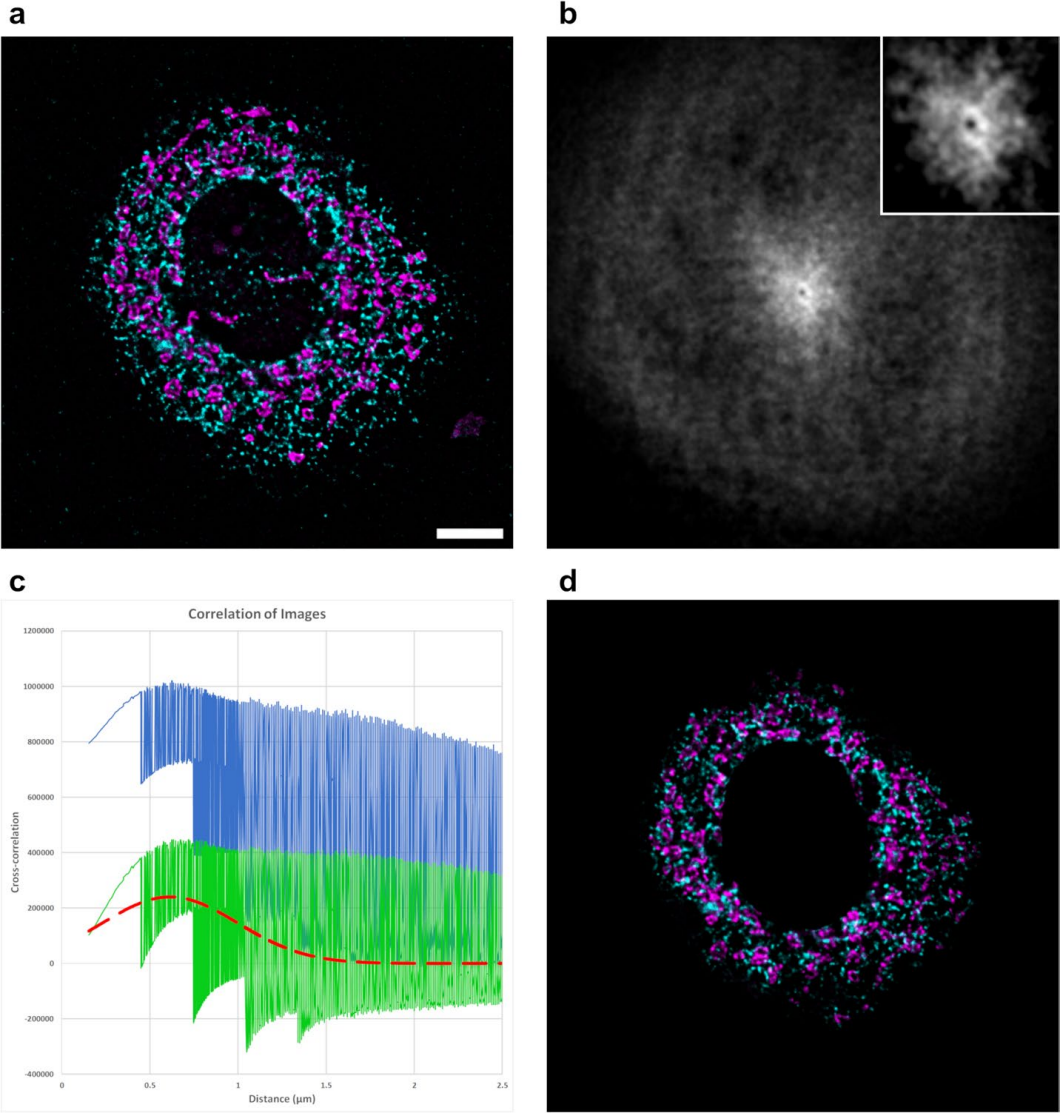
Spatial correlation of mitochondria and ER. **a** Lateral cross-section of an MLE-12 cell stained for mitochondria (magenta) and ER (cyan). Scale bar indicates 5 µm for all images. **b** Cross-correlation image following removal of low spatial frequency contributions. (inset) Contrast enhanced and enlarged image of the center of the cross-correlation image. The dim pixels at the center indicate low correlation of untranslated images. **c** The blue line is the radial profile of the original cross-correlation, the green line is the radial profile after the pixel randomization procedure (see Implementation for details), and the red dashed line is the fit Gaussian curve. **d** Composite image of the data that contributed to the Gaussian result. ER near the cell periphery or otherwise distant from mitochondria are notably dimmer. The region of the nucleus was omitted from analysis using the input mask

While artificial test data is great for testing the isolated effects of various imaging parameters on the results of any plugin, it is ultimately meaningless if the plugin fails on real-world data. To this end, cells stained for their endoplasmic reticulum and mitochondria were analyzed with CCC (Fig. 2). While the mitochondria and ER are not the traditional paired or binding structures studied via colocalization assays, these organelles must maintain a close proximity for the exchange of lipids and Ca^2+^, without allowing complete membrane fusion [24]. CCC was successfully able to identify an average distance of 0.61 µm between the two organelles, with a σ value of 0.39 µm. These values corresponded very well to the manual distance measurements (0.53 µm ± 0.17), which were acquired using the line tool between local ER and mitochondrial intensity peaks. The confidence and R^2^ values of 0.15 and 0.1, respectively, were much lower compared to the artificial data, but are within a comparable range for other real biological samples that have been tested (data not shown).

The steps present in Fig. 2c are due to anisotropy of the sample itself. Translations along the z-axis return much lower cross-correlation values than lateral translations because the cell spread out laterally on the glass surface, confining most of the ER and mitochondria to only a few lateral cross-sections. The period of the steps, 299 nm, corresponds perfectly with the voxel depth of the image.

## Discussion

CCC is a novel algorithm and ImageJ plugin for measuring the spatial correlation between two images. Unlike traditional colocalization algorithms, CCC does not require there to be overlap between the signals of the two images to find a true spatial correlation. This is becoming increasingly important in the field of bioimaging as new super-resolution microscopy methods make it increasingly unlikely that two spatially correlated proteins will have overlapping PSFs after imaging. As demonstrated in Table 1, CCC is capable of correctly identifying spatial correlations with no pixel overlap from a variety of imaging modalities, including super-resolution 3D-STED microscopy. CCC breaks the overlap requirement by using the cross-correlation function to measure the correlation as a function of distance.

While similar ICCS methodologies have been previously implemented in MATLAB [13, 15], CCC improves upon these methods in several ways. First, the MATLAB implementations are limited to 2D images, while CCC can analyze 3D images. This increases the accuracy of CCC and allows for the analysis of more complex structures than is possible with 2D data. Additionally, CCC is able to calculate the signal in each of the original images that most contributed to the resulting Gaussian curve fit. While this is common practice with pixel-wise colocalization methods, this is a novel feature for any ICCS methodology. These contribution images will enable researchers to fully understand where a spatial correlation is occurring within their cells. They can also be used to aid in troubleshooting potential issues in the images when the results are not as expected. Lastly, CCC is designed to be a more user-friendly plugin than the MATLAB implementations by limiting the number and importance of adjustable parameters, and not requiring the creation of complex scripts.

A significant advantage of all ICCS methods is that various image quality metrics (resolution, background, noise, autocorrelation, etc.) are inherently integrated into the results, even when the signal from the two images overlap and produce µ values of zero. As demonstrated in nearly all the tables, using better imaging techniques and microscope objectives, and working to improve the SNR, will improve the accuracy, precision, and statistical parameters of the CCC plugin. This is in sharp contrast to traditional pixel-wise colocalization methods, such as PCC, where differences in image resolution and quality are generally not reflected in the results, and efforts to improve resolution can make the results worse as the overlap between the PSFs is reduced.

The statistical parameters provided by CCC have not been used in any other colocalization algorithms. While R^2^ is a commonly reported parameter for curve fitting, its implication in CCC is unique. As can be seen in Table 7, R^2^ values lower with increasing noise. However, what is less transparent, is that the degree of anisotropy of both the PSF and the sample influence R^2^ as well. Since the axial resolution of most imaging systems is less than the lateral resolution, substantially different correlation values can be obtained for very similar distances depending on the direction of data translation, leading to spiky data (Fig. 2c) and lower R^2^ values. This can be compounded by anisotropy of the sample itself, as is the case with broad flat cells (Fig. 2). Generally speaking, image noise and PSF anisotropy should not be great enough to cast doubt into the results of CCC, except in extreme cases. However, low R^2^ values can, in rare cases, be indicative of an improper curve fitting to a dataset that does not have any spatial correlation.

The confidence value reported by CCC is wholly unique and is the primary statistical metric for the plugin. Other colocalization algorithms attempt to compare their results to a null hypothesis image dataset for statistical measures. However, for various reasons, most of these methods have been found to be misleading or inappropriate [16, 25]. Instead of comparing to a null hypothesis, confidence is simply a metric that reflects numerous sample and image parameters that lead to uncertainty in colocalization results. Low confidence could result from any combination of high autocorrelation in one or both images, either due to: 1) low resolution (Tables 1, 2) or very high molecular density (Table 4), 2) a large SCD relative to the resolution (Table 3), or 3) a low ratio of correlated to uncorrelated particles (Table 5). Additionally, while image background will inappropriately lead to low confidence, background can be easily removed during pre-processing (see Implementation).

Since confidence is a novel metric, it has no precedence for a value threshold that would indicate a likely true positive correlation, and the values from the tables presented here are unnaturally high due to their ideal conditions. From what biological data has been analyzed using CCC, it is estimated that a confidence value of 0.1 or greater indicates a reasonably likely true correlation, and values of 0.2 or greater indicating a very likely true correlation. Should CCC results be consistent across experimental repeats, but with confidence values below 0.1, efforts should be taken to improve the quality of the input images. Such efforts could be as simple as ensuring that the pre-processing steps have been completed appropriately, or performing deconvolution on the input images. It could also mean altering the sample preparation protocol to reduce non-specific staining, or that a higher-resolution imaging system is needed.

When reporting results from CCC it is important to always report the spatial correlation with both the µ and σ values. A greater emphasis was placed on the accuracy of the µ value for the artificial test data results in the tables above, but this could only be done because the true SCD was a known value. For research applications, the SCD will be unknown, and the spatial correlation should always be discussed as being within a range defined by µ and σ. Reporting the σ value is particularly important for µ values of zero, as µ = 0 implies that the imaging system had insufficient resolution to measure the true SCD, which cannot be zero as no two physical particles can occupy the same space. Likewise, when statistically comparing spatial correlation results between experimental conditions, it is important to include both parameters by performing a Hotelling’s T^2^ test [26], or a multivariate analysis of variance (MANOVA) [27].

A persistent phenomenon in the test data presented was that the µ value returned was consistently lower than the true SCD. This is not a coincidence and is due to a specific and unavoidable mathematical effect from the plugin. Further details on this can be found in Additional file 1.

Throughout this manuscript, the applicability of CCC towards super-resolution 3D biological fluorescent images has been the primary focus. However, CCC is broadly applicable to 2D or 3D image data of any resolution from potentially any field. In any case where the spatial correlation or average distance between two image components needs to be measured, and those components can be isolated into two images, CCC could be applied. While CCC assumes and fits a Gaussian curve to the data, it also provides a table of all the cross-correlation values used for this process. This allows the user to fit their own model to their data, as well as determine if a second correlation exists beyond the CCC result. This further allows CCC to be used to measure average distances between a single component of an image (e.g. the average distance between nuclei) by performing an autocorrelation with CCC and fitting a curve to the first peak beyond a distance of zero.

Additionally, CCC is capable of directly handling time-series data. The plugin analyzes each frame of the image dataset individually, producing the same results shown in the tables for every time point. The radial profile data for each time point is aggregated into a new image and presented as a heatmap (see Additional file 1: Figure S4), and the frame with the highest confidence value is directly reported. This feature makes it much easier to study transient or dynamic processes, particularly changes to spatial correlation that may occur in response to an external stimulus, such as ligand stimulation or environmental changes.

Though CCC has many strengths, there are still a few ways that the algorithm could be improved. First, the runtime of the plugin could be improved. At the moment, analysis of large volumes in CCC takes a significant amount of processing time and memory (see Additional file 1 for more details). While it is not clear if further memory optimizations will be possible, the runtime of CCC will be improved in future updates.

Another area of potential improvement is within the pixel randomization step. CCC uses pixel randomization during the calculation of confidence and as a method of removing undesired low-spatial frequency contributions. Currently, this step randomizes one of the input images a number of times specified by the user (see Additional file 1 for discussion on selecting the number of iterations). This randomization could be performed on both images sequentially instead of just one image, increasing the robustness of the algorithm. Implementing this adds a very significant cost to the processing speed and is beneficial only in instances where the two images have notably differing spatial distributions or densities, and is planned for after further performance optimizations. Additionally, it may be possible to automatically determine the number of randomization iterations to perform instead of using a user-set value. This would make the plugin less user-error prone and simplify its execution.

## Conclusion

In summary, CCC offers many unique advantages over other colocalization methods and ICCS implementations. Its improved compatibility with 3D super-resolution imaging makes it more applicable to modern image datasets. The reported parameters (µ, σ, confidence, and R^2^) provide a more complete picture of the relation between the two images, the image quality, and what could be improved to obtain better results. Lastly, the ability to identify the signal in each image that most contributed to the results will aid the researcher in understanding the role a spatial correlation plays in their research.

## Availability and requirements

Project name: Colocalization by Cross Correlation.

Project home page: https://imagej.net/plugins/colocalization-by-cross-correlation.

Operating systems: Platform independent.

Programming Language: Java.

Other requirements: Fiji release 2.14.0 or higher.

License: GPLv3.

Any restrictions to use by non-academics: Those defined in the GPLv3 license.

### Supplementary Information

**Additional file 1**. Supplemental discussions, and supplementary figures 1, 2, 3, and 4.

**Additional file 2**. A 3D projection of a test input image with 50 spatially correlated pairings at 1.13 μm correlation distance, and created using deconvolved SD-40 PSFs collected with a 40×/1.3 objective.

**Additional file 3**. The ImageJ macros used to generate the test input images.

## Data Availability

Data is available at https://buffalo.box.com/s/gp4u55wt2lykpr6nm78sy998ngako878. Source code for CCC is available at https://github.com/andmccall/Colocalization_by_Cross_Correlation. The ImageJ macros used for data generation are available in Additional file 3.

## Abbreviations

CCC: Colocalization by cross-correlation plugin
CCF: Cross-correlation function
ICCS: Imaging cross-correlation spectroscopy
PCC: Pearson’s correlation coefficient
µ: The mean of the Gaussian curve fit by CCC
σ: The standard deviation of the Gaussian curve fit by CCC
SCD: Spatial correlation distance
SNR: Signal to added noise ratio
WF: Widefield microscopy
SD: Spinning disk microscopy
STED: Stimulated emission depletion microscopy
PSF: Point spread function
CCR: Cross-correlation result
SCCR: Subtracted cross-correlation result
GCCR: Gaussian-modified cross-correlation result
