# Supplementary material for: Colocalization by cross-correlation, a new method of colocalization suited for super-resolution microscopy
Source: BMC Bioinformatics. 2024 Feb 2;25:55. doi: 10.1186/s12859-024-05675-z (PMC10837882; doi:10.1186/s12859-024-05675-z)
Supplement: Supplementary file 1 — Additional file 1. Supplemental discussions, and supplementary figures 1, 2, 3, and 4. [file 12859_2024_5675_MOESM1_ESM.pdf]

Supplementary notes

ImgLib2 cross-correlation algorithm

ImgLib2 calculates the cross-correlation of two images through the utilization of the cross-correlation theorem, which states that the cross-correlation of two signals in the spatial domain is equal to their pointwise product in the frequency domain. To start, ImgLib2 first computes the fast Fourier transform (FFT) of both images, converting both images to their frequency domain counterparts. Importantly, when transforming the second image, the complex conjugate of the image is calculated, which is what distinguishes the cross-correlation calculation from convolution. Then, the pixel-wise product of these FFT images is calculated and the result is inverse transformed back into the spatial domain, producing a new image of the cross-correlation result. This process is much faster compared to calculating the cross-correlation by iterating over every possible transformation between two images in the spatial domain, which is extremely slow.

Cause of shorter μ

The shorter μ value compared to the true SCD that is present throughout the results comes from the calculation of the radial profile. Since the radial profile takes the average correlation at any given distance, it can be viewed as taking the average across the surface area of concentric spheres. Thus, the number of pixels over which we are averaging increases at a rate of roughly the distance squared as we profile greater distances. This results in the data being slightly skewed towards zero and is why our μ value results are consistently lower.

Another ICCS algorithm dealt with this by using an empirically determined correction factor that was adjusted dependent upon the lateral full-width at half maximum (FWHM) of the point spread function (PSF)[1]. However, with 3D ICCS like in CCC, the correction factor would need to be dependent upon both the lateral and axial FWHM, and the contribution of each FWHM value to the correction factor would depend on the average orientation of the spatial correlation. For example, cultured cells grown on a coverslip would be much more laterally oriented, and thus more dependent on the lateral FWHM, than an ex vivo organoid. Thus, it would be very complicated to implement a correction factor using FWHM values in CCC and would add numerous additional parameters that need to be set by the user.

Discussion on memory and runtime

CCC demands considerable computational resources during its execution. As an example, CCC analysis of two 1 GB 32-bit format images requires approximately 45 GB of memory. These values include the calculation of the contribution images, 10 randomization cycles, and no mask provided. Thus, in terms of memory, CCC requires roughly 50 times as much available memory as the 32-bit format size of one of the input images, which is consistent with other image sizes. Since all the calculations performed in CCC require floating-point precision, which is accomplished using 32-bit formats, providing 8 or 16-bit images to CCC makes a negligible difference in the memory requirements. Cross-correlation is also not a task that can be split up into smaller parts and produce the same result, limiting room for memory optimization. Importantly, since each frame of time-lapse data is analyzed individually, the memory requirements for time-lapse datasets would only be a little over 50 times the size of a single frame. Some additional memory would be required as the output is consolidated.

Runtime speed is much more variable, as it is highly dependent on the specifications of the computer, but for the example above, with an Intel Core i7-8700 3.2GHz CPU, the runtime was 80 minutes. However, this could have been substantially improved if the computer had more available memory, as its 52 GB was near the minimum requirement to analyze these images. Being near the minimum causes the Java garbage collector to run frequently and increases runtime significantly. Currently, CCC is multi-threaded optimized, so increasing the number of logical processors can also improve the runtime. The runtime speed of CCC could be further improved with a GPU-optimized cross-correlation algorithm, something that is currently in development.

Determining the number of iterations to use

Currently, the number of randomization iterations is a value that must be set by the user for CCC analysis. Increasing the number of iterations generates more consistent results, primarily resulting in less variation in the σ and confidence values, but also increases computation time significantly. To determine a minimally appropriate number of iterations the user can simply analyze the same two images multiple times for various randomization iterations, and compare the standard deviation or range of the confidence values. As an example, when the deconvolved SD-40, 1.13 µm spatial correlation distance test image is analyzed in this way, 10 repeat analyses using only a single randomization step results in confidence ranging from <> to <>. Using the same process and images with 10 randomization steps for each analysis results in confidence ranging from <> to <>, substantially less variation. These results will change depending on the characteristics input images (molecular density, fluorophore distribution, etc.), so should be tested for any new research project. In general, most datasets do not require more than a few iterations to get to quite low variability in the results. Ten iterations was used in this manuscript to ensure that the observed variability was due to real differences in the test images, and not due to variability from the randomization process. However, for most experimental applications, this number of iterations would probably be unnecessary.

References

1. Oneto, M. *et al.* Nanoscale distribution of nuclear sites by super-resolved image cross-correlation spectroscopy. *Biophysical journal* **117**, 2054–2065 (2019).

Supplementary Figures

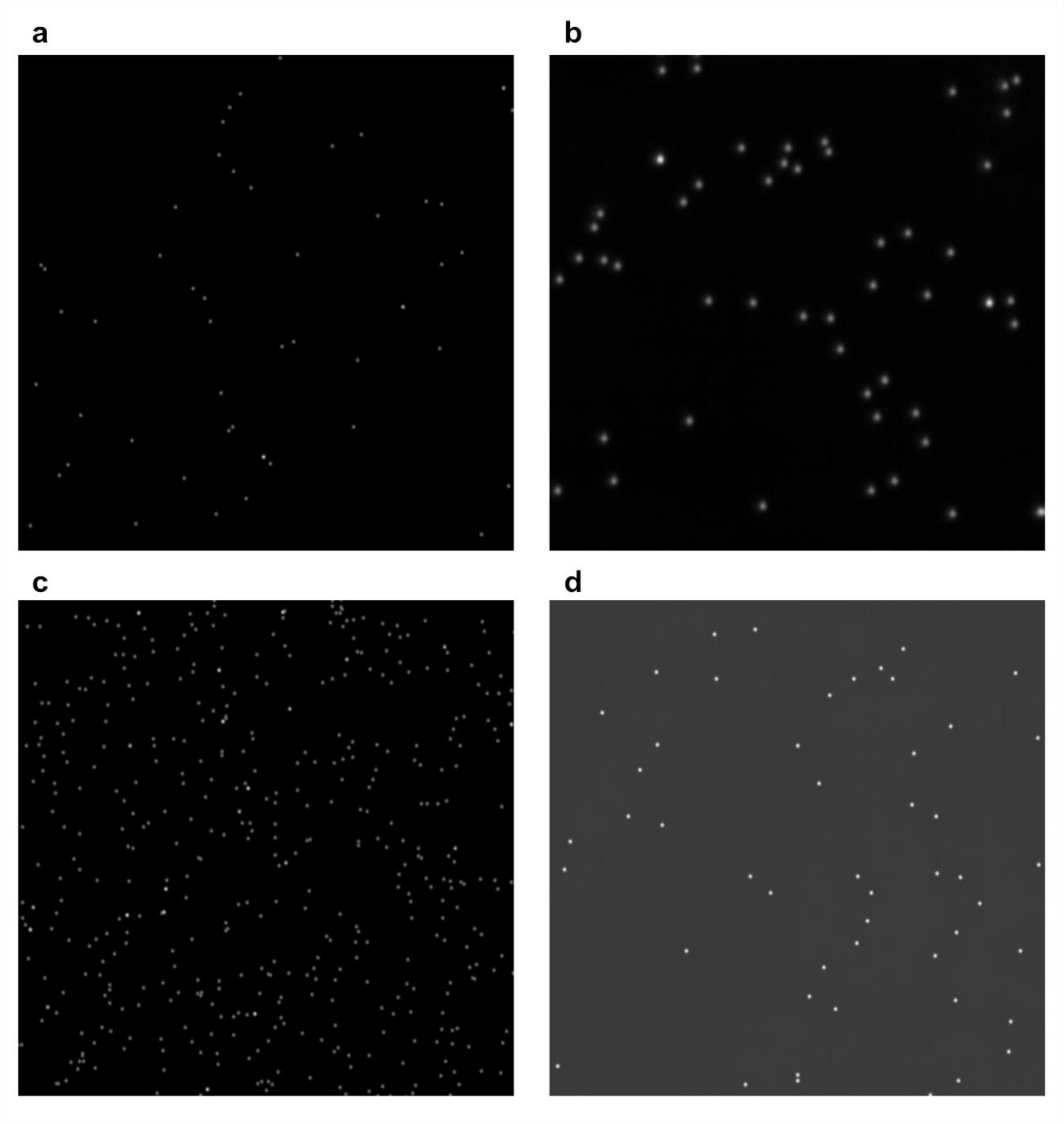

Supplementary Figure 1. Examples of various causes of autocorrelation. Z-projections of test images that demonstrate various levels of autocorrelation. (**a**) A comparatively low autocorrelation image with an autocorrelation of 4.82×10^{13}. (**b**) An image with higher autocorrelation, 1.23×10^{14}, due to lower resolution. (**c**) An image with higher autocorrelation, 2.51×10^{15}, due to high molecular density. (**d**) An image with higher autocorrelation, 5.32×10^{14}, due to high background. The autocorrelation value was measured as the area under the curve of the radial profile of the autocorrelation image.

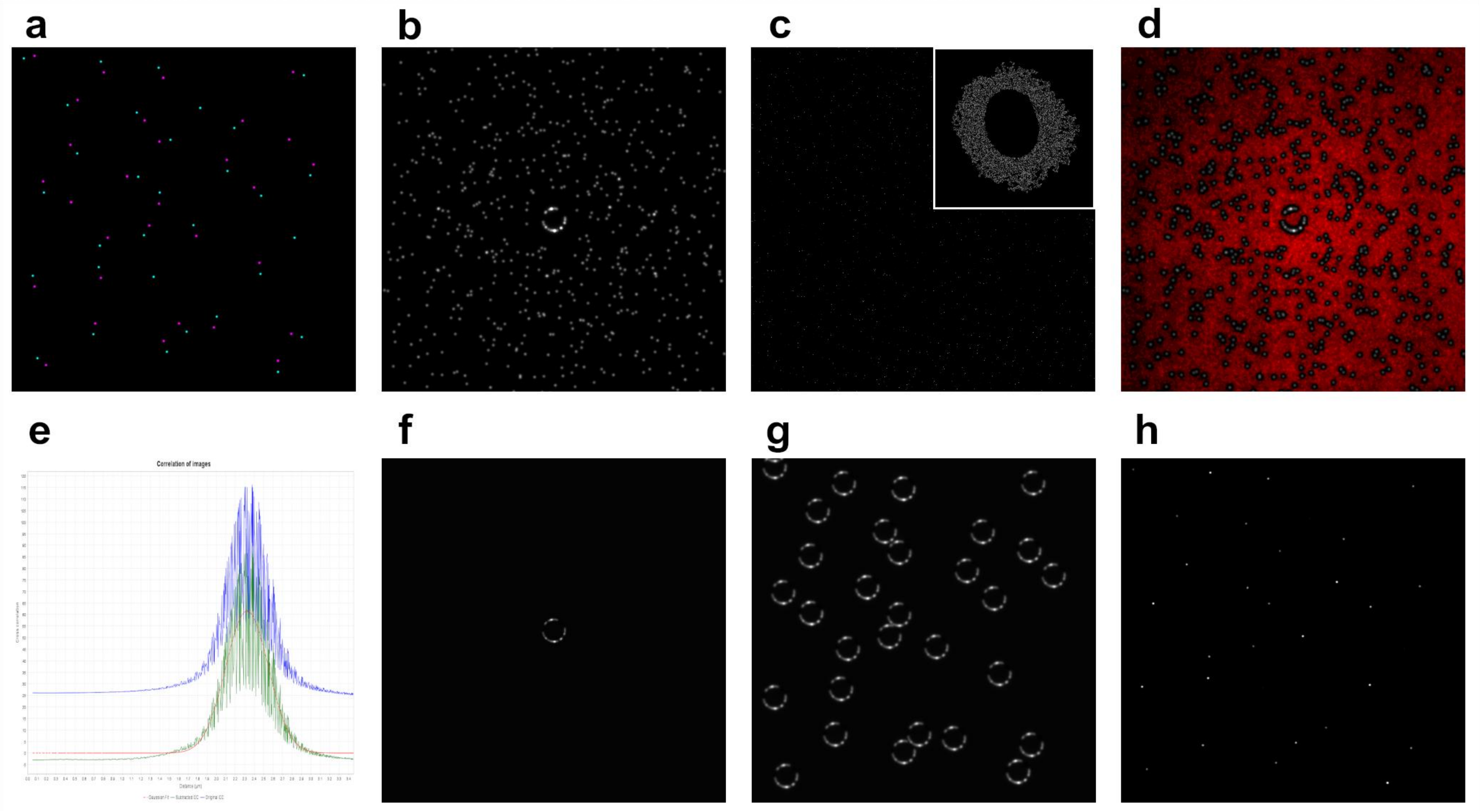

Supplementary Figure 2. Graphical Abstract. A full overview of CCC processing. (**a**) Color composite of the two input images, with most of the magenta points being paired to a corresponding cyan point. (**b**) The image generated from the cross-correlation (CC) of the input images. (**c**) A pixel randomized image of the cyan input image. Multiple randomized images are cross-correlated with the magenta image and the average CC is taken for the next step. (**c - inset**) A pixel randomized image of the cell from Figure 2, demonstrating randomization within the mask. (**d**) The average CC result from the randomization step is subtracted from the original CC result (b) to generate this subtracted CC image. In this case the image is nearly identical because we are randomizing with no mask, but all the values are lower. Red indicates negative values. (**e**) The radial profile plot of (b) in blue and (d) in green. A Gaussian curve (red) is then fit to the green line to determine the spatial correlation. (**f**) To start the process of generating the contribution images the Gaussian curve fit from (e) was used to modify the SCCR image (d) to generate this Gaussian-modified CC image, suppressing correlations outside of the curve fit. (**g**) The result of convolving the Gaussian-modified CC image with the magenta colored input image from (a). The rings are all the points that are ~1 µ away (2.32 µm) from a magenta PSF. (**h**) The (g) image is then pixelwise multiplied with the cyan input image to generate the cyan contribution image. All steps in this figure except (g) can be generated as output from CCC.

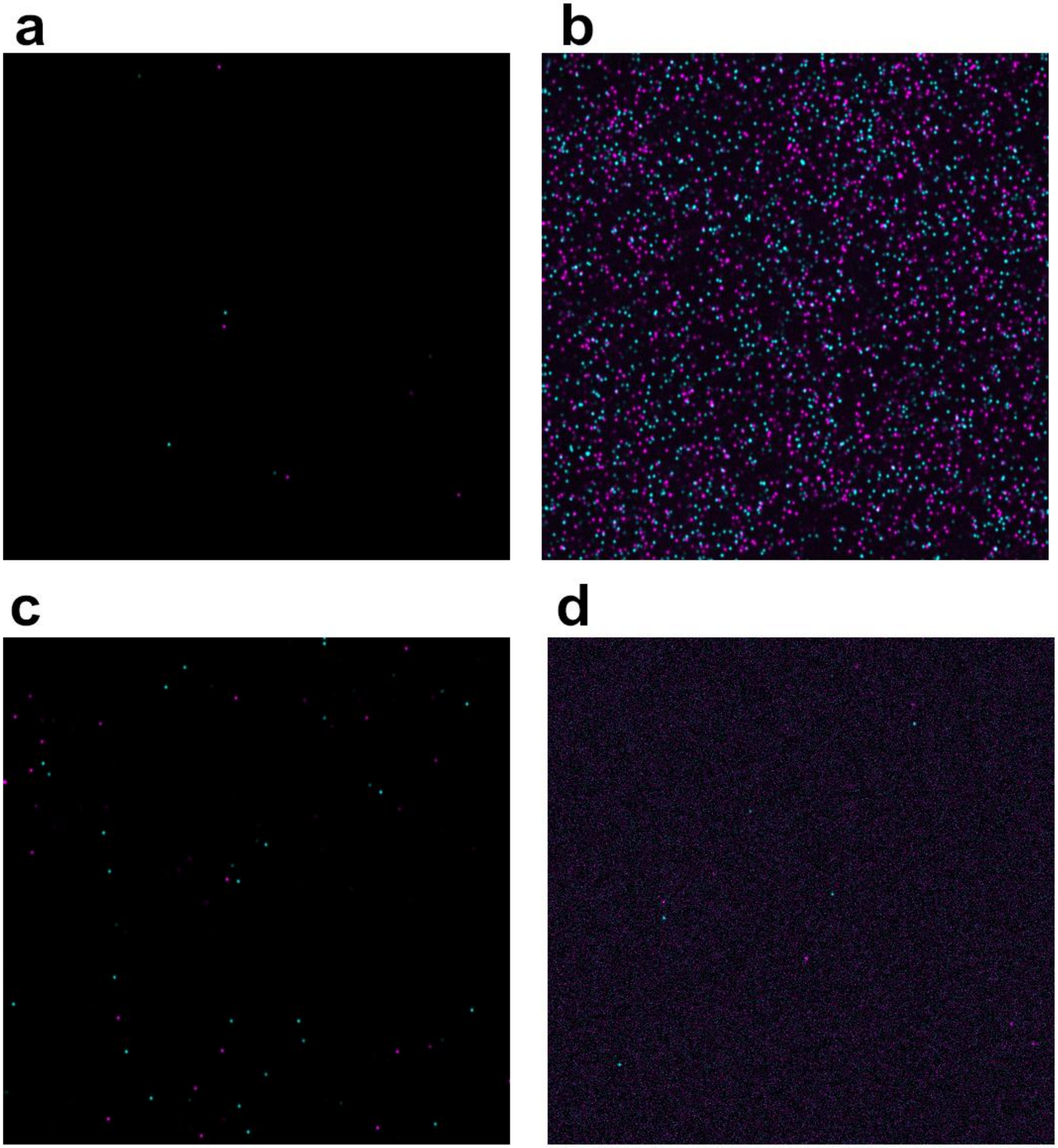

Supplementary Figure 3. Examples of various test images. Lateral cross-sections of 3D test images used in the manuscript. Cross-sections with at least one lateral magenta-cyan pair at 1.13 µm were chosen. (**a**) An example of the base test image used in all tables. Deconvolved SD-40 PSFs imaged with a 40x/1.3NA objective. Has a paired particle density of $3.48 \times 10^{-4}/\mu m^3$ with no additional noise or background, and no unpaired particles. (**b**) An example test image from Table 4 with a paired particle density of $1.74/\mu m^3$ and no unpaired particles. (**c**) An example test image from Table 5 with additional unpaired/non-correlated particle density of $2.79 \times 10^{-2}/\mu m^3$ for each channel. Paired particle density is $3.48 \times 10^{-4}/\mu m^3$. (**d**) An example test image from Table 7 with a signal to additional electronic noise ratio of 20:1 for both channels.

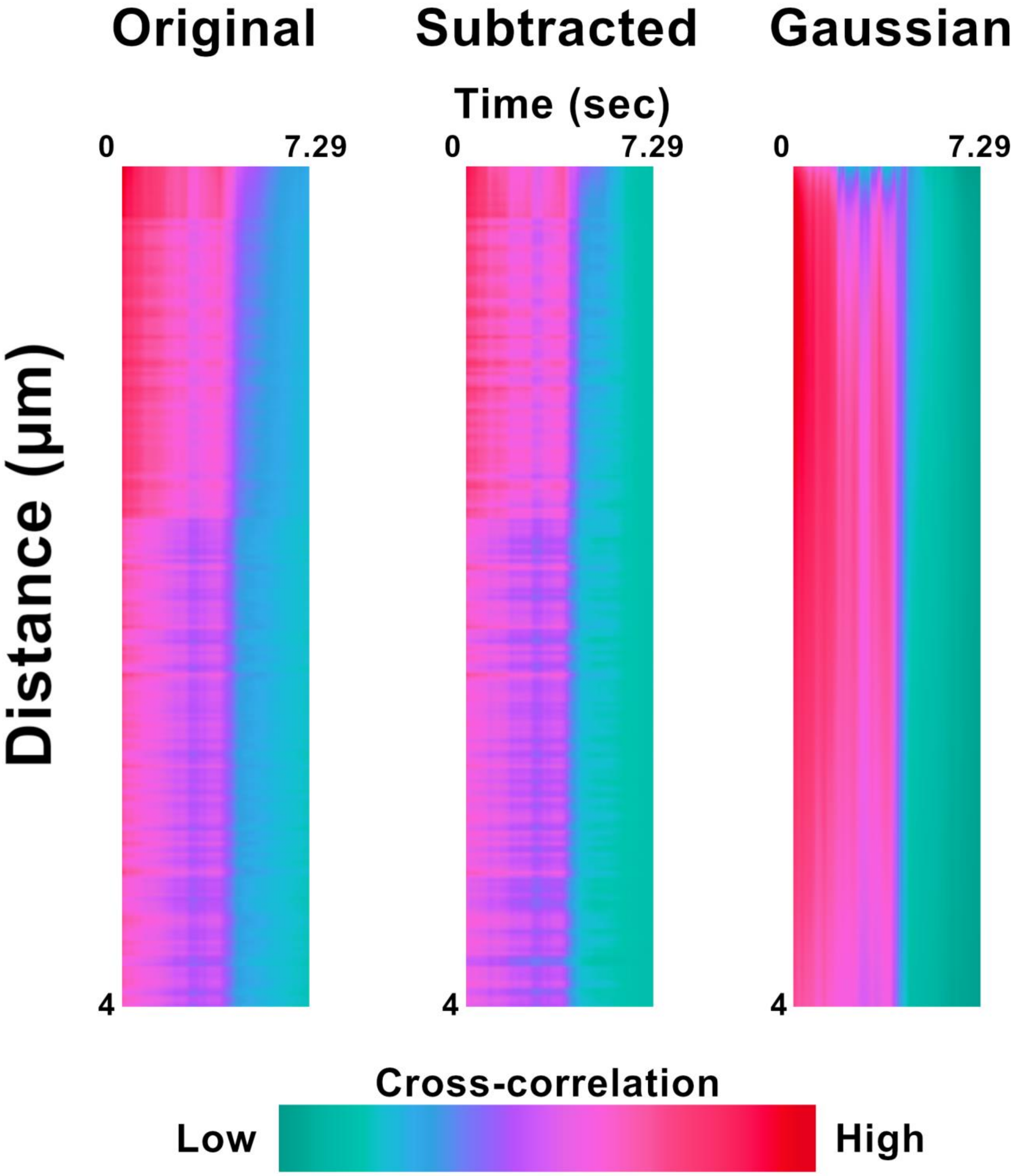

Supplementary Figure 4. Example of CCC output for time-lapse data. Heatmaps of the radial profiles of the original cross-correlation, the cross-correlation after subtraction of low-frequency contributions, and the Gaussian fit curves of time-lapse image data. For each heatmap, every column represents the radial profile at that time point, and every row a distance. This example data was generated from the mitosis sample dataset included with Fiji. Intensity scaling is not consistent between the heatmaps. The decrease in cross-correlation starting around 4 seconds corresponds to the cell entering anaphase.
